# Supplementary material for: Sperm chemotaxis is driven by the slope of the chemoattractant concentration field
Source: eLife. 2020 Mar 9;9:e50532. doi: 10.7554/eLife.50532 (PMC7093112; doi:10.7554/eLife.50532)
Supplement: Supplementary file 1. — Note that the main differences between species are the number of receptors N. N1/2 number of receptors that allows half maximal binding rate for any concentration of chemoattractant, that is πa/s. D diffusion coefficient of the chemoattractant; Kon association rate constant; s effective radius of the chemoattractant (as proxy of chemoattractant receptor's binding site radius); Δt sampling time (time to swim half the circumference in the boundary close to the water-glass interface); v mean linear speed of the spermatozoa, Δr/Δt; Δr sampling distance (circumference diameter); L length of sperm flagellum; a spermatozoa radius, assuming that flagella are spheres; Pe Peclet number for a spherical cell approximation (sphere), or cylindrical flagellum geometry (cylinder). a Measured in this study (mean ± sd); N = 3 sea urchins; n = 495 (S. purpuratus), n = 56 (L. pictus) spermatozoa. bNishigaki and Darszon (2000); Nishigaki et al. (2001). c Calculated in this study (see section 1.1. On the estimate of maximal chemoattractant absorption). d Measured in this study (mean ± sd); N = 1 sea urchin; n = 26 (S. purpuratus), n = 39 (L. pictus) spermatozoa. eKashikar et al. (2012). fPichlo et al. (2014) reported 6.5 x 10−8 cm for the resact radius. g Calculated in this study. [file elife-50532-supp1.docx]

|  | ***S. purpuratus*** | ***L. pictus*** | ***A. punctulata*** |
| --- | --- | --- | --- |
| $\boldsymbol{N}$ **[per cell]** | 2.0 x 10^4 b^ | 6.3 x 10^4 b^ | 3.0 x 10^5 f^ |
| $\boldsymbol{N}_{\boldsymbol{1/2}}$ | 2.9 x 10^4 c^ | 3.6 x 10^4 c^ | 1.8 x 10^4 c^ |
| $\boldsymbol{D}$ **[cm^2^ s^-1^]** | 2.4 x 10^-6 c^ | 2.4 x 10^-6 c^ | 2.4 x 10^-6 e^ |
| ***Kon* [M^-1^ s^-1^]** | 2.7 x 10^7 b^ | 2.4 x 10^7 b^ | 5.0 x 10^7 f^ |
| $\boldsymbol{s}$ **[cm]** | 1.87 x 10^-8 c^ | 1.66 x 10^-8 c^ | 3.46 x 10^-8 c^ |
| $\boldsymbol{\Delta}\mathbf{t}$ **[s]** | 0.39 ± 0.08 ^a^ | 0.52 ± 0.22 ^a^ | 0.60 |
| $\boldsymbol{v}$ **[cm s^-1^]** | 71.8 x 10^-4 c^ | 88.5 x 10^-4 c^ | 100 x 10^-4 c^ |
| $\boldsymbol{\Delta}$***r* [cm]** | 28 ± 6 x 10^-4 a^ | 46 ± 14 x 10^-4 a^ | 60 x 10^-4^ |
| ***L* [cm]** | 39.2 ± 2.2 x 10^-4 d^ | 48.7 ± 2.1 x 10^-4 d^ | 50 x 10^-4 e^ |
| ***a* [cm]** | 1.39 x 10^-4 c^ | 1.56 x 10^-4 c^ | 1.58 x 10^-4 c^ |
| ***Pe*** | 4.2 x 10^-1^ (sphere)  6.0 x 10^-2^ (cylinder) ^g^ | 5.8 x 10^-1^ (sphere)  7.4 x 10^-2^ (cylinder) ^g^ | 6.6 x 10^-1^ (sphere)  8.3 x 10^-2^ (cylinder) ^g^ |

**Supplementary File 1**

**Parameters of the chemoattractant sampling model for each species.**

Note that the main differences between species are the number of receptors $N$. $N_{1/2}$ number of receptors that allows half maximal binding rate for any concentration of chemoattractant, i.e. π*a/s*. *D* diffusion coefficient of the chemoattractant; $Kon$ association rate constant; $s$ effective radius of the chemoattractant (as proxy of chemoattractant receptor's binding site radius); $\Delta t$ sampling time (time to swim half the circumference in the boundary close to the water-glass interface); *v* mean linear speed of the spermatozoa, i.e.$\Delta$*r/*$\Delta t$; $\Delta r$ sampling distance (circumference diameter); *L* length of sperm flagellum; *a* spermatozoa radius, assuming that flagella are spheres; *Pe* Peclet number for a spherical cell approximation (sphere), or cylindrical flagellum geometry (cylinder). ^a^ Measured in this study (mean ± sd); N = 3 sea urchins; n = 495 (*S. purpuratus*), n = 56 (*L. pictus*) spermatozoa. ^b^ Nishigaki et al., 2001; Nishigaki and Darszon, 2000. ^c^ Calculated in this study (see section ***1.1. On the estimate of maximal chemoattractant absorption***). ^d^ Measured in this study (mean ± sd); N = 1 sea urchin; n = 26 (*S. purpuratus*), n = 39 (*L. pictus*) spermatozoa. ^e^ Kashikar et al., 2012. ^f^ Pichlo et al., 2014 reported 6.5 x 10^-8^ cm for the resact radius. ^g^ Calculated in this study.
