# Supplementary material for: Sperm chemotaxis is driven by the slope of the chemoattractant concentration field
Source: eLife. 2020 Mar 9;9:e50532. doi: 10.7554/eLife.50532 (PMC7093112; doi:10.7554/eLife.50532)
Supplement: Supplementary file 2. — *Typically, there is an extra 20% loss of light power between the back focal plane of the objective and the sample, due to scattering within the optics. [file elife-50532-supp2.docx]

|  | Physical diameter (mm) | UV power at the back focal plane of the objective (mW)* |
| --- | --- | --- |
| ***f1*** | 0.2 | 0.07 |
| ***f2*** | 0.6 | 1.25 |
| ***f3*** | 2 | 4.7 |
| ***f4*** | 4 | 7.8 |
| ***f5*** | 4 | 9.46 |

**Supplementary File 2**

**Physical diameter of the optical fibers, and UV light power measured at the back focal plane of the objective.**

*Typically, there is an extra 20% loss of light power between the back focal plane of the objective and the sample, due to scattering within the optics.
