# Supplementary material for: Sperm chemotaxis is driven by the slope of the chemoattractant concentration field
Source: eLife. 2020 Mar 9;9:e50532. doi: 10.7554/eLife.50532 (PMC7093112; doi:10.7554/eLife.50532)
Supplement: Transparent reporting form [file elife-50532-transrepform.docx]

***eLife’s* transparent reporting form**

We encourage authors to provide detailed information *within their submission* to facilitate the interpretation and replication of experiments. Authors can upload supporting documentation to indicate the use of appropriate reporting guidelines for health-related research (see [EQUATOR Network](http://www.equator-network.org/%20)), life science research (see the [BioSharing Information Resource](https://biosharing.org/)), or the [ARRIVE guidelines](http://www.plosbiology.org/article/info:doi/10.1371/journal.pbio.1000412) for reporting work involving animal research. Where applicable, authors should refer to any relevant reporting standards documents in this form.

If you have any questions, please consult our Journal Policies and/or contact us: [editorial@elifesciences.org](mailto:editorial@elifesciences.org).

**Sample-size estimation**

- You should state whether an appropriate sample size was computed when the study was being designed
- You should state the statistical method of sample size computation and any required assumptions
- If no explicit power analysis was used, you should describe how you decided what sample (replicate) size (number) to use

Please outline where this information can be found within the submission (e.g., sections or figure legends), or explain why this information doesn’t apply to your submission:

*We did not use any statistical inference to estimate the size of the experimental sample. The minimal sample size was defined based on our previous studies of sperm chemotaxis of marine invertebrates (Guerrero et. Al, Dev Cell; 2010). Briefly, each cell was considered as an experimental replicate (20- 50 cells per video) for single-cell analysis, which was obtained from a video-microscopy recording of swimming spermatozoa (20 – 70 replicates per experimental condition).*

*At least 3 different sea urchins were used for each experimental condition. Five distinct experimental regimes were tested (spermatozoa exposed to five distinct chemoattractant concentration regimes).*

*The number of population experiments (N) can be consulted in Figure 3b, and the number of sperm analyzed in single-cell analysis (n) can be consulted in Figure 4d.*

**Replicates**

- You should report how often each experiment was performed
- You should include a definition of biological versus technical replication
- The data obtained should be provided and sufficient information should be provided to indicate the number of independent biological and/or technical replicates
- If you encountered any outliers, you should describe how these were handled
- Criteria for exclusion/inclusion of data should be clearly stated
- High-throughput sequence data should be uploaded before submission, with a private link for reviewers provided (these are available from both GEO and ArrayExpress)

Please outline where this information can be found within the submission (e.g., sections or figure legends), or explain why this information doesn’t apply to your submission:

*Reproduction of sea urchins is seasonal. The reproductive season of S. purpuratus species in the coastal areas of California and Baja California lasts 4-7 months (autumn-winter). Preliminary experiments were performed in 2007, formal experimental studies were done in the course of a single month (July-August 2008). Data mining and modeling was performed between 2009 and 2018.*

*In this work, spermatozoa of distinct males were considered as biological replicates, and video-microscopy recordings as technical replicates.*

*Figures 1 and Figure 1-figure supplement 1 correspond to theoretical studies (mostly modeling), hence they lack experimental replicates. The chemoreception models were parameterized with the data presented in Supplementary File 1, which contains calculations derived from experimental data.*

*Figures 2, 3, Figure 3-figure supplement 1 and Figure 3-figure supplement 2 present studies at the population level of swimming spermatozoa. The data include the pooled analysis of more than three sea urchins. In total,* *267 video-microscopy recordings were computed: 20 of them were for the f1 fiber, 71 for f2, 38 for f3, 44 for f4, 18 for f5, 46 for the HighK^+^ control and 30 for the Low Ca^2+^ control. The automatized analysis of swimming spermatozoa is described on section: “Quantitation of global changes of spermatozoa number and [Ca^2+^]_i_”. These replicates can be consulted in Figure 3b.*

*Figures 4, 5, 6, Figure 3-figure supplement 3, Figure 4-figure supplement 1, Figure 5-figure supplement 1, Figure 5-figure supplement 2, Figure 6-figure supplement 1 and Figure 6-figure supplement 2. Single cell analysis of swimming spermatozoa. Almost 1000 single spermatozoa exposed to five distinct speract concentration gradients were manually tracked and analyzed. The data include the pooled analysis of up to 3 sea urchins. Former description of the analysis, alongside the description of experimental replicates is presented on sections: “Computing the dynamics of speract concentration gradients”, and “Computing [Ca^2+^]i dynamics and the stimulus function of single spermatozoa”. These replicates can be consulted in Figure 4d.*

*Outliers were not excluded in any way in this study.*

*There was no data omission/exclusion of any type in this work.*

**Statistical reporting**

- Statistical analysis methods should be described and justified
- Raw data should be presented in figures whenever informative to do so (typically when N per group is less than 10)
- For each experiment, you should identify the statistical tests used, exact values of N, definitions of center, methods of multiple test correction, and dispersion and precision measures (e.g., mean, median, SD, SEM, confidence intervals; and, for the major substantive results, a measure of effect size (e.g., Pearson's r, Cohen's d)
- Report exact p-values wherever possible alongside the summary statistics and 95% confidence intervals. These should be reported for all key questions and not only when the p-value is less than 0.05.

Please outline where this information can be found within the submission (e.g., sections or figure legends), or explain why this information doesn’t apply to your submission:

*All statistical tests were performed using R software (R Core Team, 2016). The significance level was set to p < 0.05 or p < 0.01, as indicated in each figure.*

*We have performed normality tests for all the distributions, and based on that, non-parametric analysis (boxplots, Kruskal-Wallis test, Mann-Whitney test, among others) were mainly used for null hypothesis testing. To compare two distributions we used Mann-Whitney or the t-student test; and to compare between multiple distributions we used the Kruskal-Wallis or Anova tests, for non-normal and normal distributions, respectively.*

*All statistical tests, exact values of N, definitions of center, and dispersion and precision measures used are expressed in the corresponding figure legends.*

(For large datasets, or papers with a very large number of statistical tests, you may upload a single table file with tests, Ns, etc., with reference to sections in the manuscript.)

**Group allocation**

- Indicate how samples were allocated into experimental groups (in the case of clinical studies, please specify allocation to treatment method); if randomization was used, please also state if restricted randomization was applied
- Indicate if masking was used during group allocation, data collection and/or data analysis

Please outline where this information can be found within the submission (e.g., sections or figure legends), or explain why this information doesn’t apply to your submission:

*Sea urchins were chosen randomly from our aquarium once they arrived to the laboratory. Sperm from each sea urchin were exposed to the different experimental conditions of this study.*

**Additional data files (“source data”)**

- We encourage you to upload relevant additional data files, such as numerical data that are represented as a graph in a figure, or as a summary table
- Where provided, these should be in the most useful format, and they can be uploaded as “Source data” files linked to a main figure or table
- Include model definition files including the full list of parameters used
- Include code used for data analysis (e.g., R, MatLab)
- Avoid stating that data files are “available upon request”

Please indicate the figures or tables for which source data files have been provided:

*The vast majority of the data mining presented in this work was performed through the development of several scripts/codes written in R or C/C++; which were used for image analysis (segmentation), data mining, hypothesis testing, and drawing figures.*

*All the authors agree to share the codes/programs, and source data generated alongside the entire pipeline of analysis (or at least the most relevant tables used to build the manuscript figures). We have been working on their presentation to achieve the most useful format, with the aim to facilitate further data mining. However, at this point we cannot provide them because their preparation for plain open source usage is not ready (particularly because the scripts/codes have thousands of lines that we want to comment for better understanding). We would like to ask the editors/reviewers to review the present manuscript as it is, and ask for particular data set (or codes) which might strength the scientific content of this research.*

*All the models presented include the full list of parameters used (provided in the corresponding figure legend).*
